# P055 Preventable sight loss in giant cell arteritis: bilateral temporal halo on fast-track ultrasound and the bone-protection dilemma of a prior atypical femoral fracture

**DOI:** 10.1093/rap/rkag113.085

**Published:** 2026-11-03

**Authors:** Azizi Sheik-Ali, Resmy Suresh

**Affiliations:** Croydon University Hospital, Croydon, United Kingdom; Croydon University Hospital, Croydon, United Kingdom

## Abstract

**Introduction:**

Giant cell arteritis (GCA) is the commonest systemic vasculitis of older adults and a leading cause of preventable blindness. Visual loss is usually irreversible, so immediate high-dose glucocorticoids aim to protect the fellow eye rather than restore it. We present an 86-year-old woman in whom the diagnosis was reached only after several points of contact, by which time monocular blindness was irreversible. The case highlights three teaching points: early recognition to prevent sight loss; fast-track ultrasound securing the diagnosis despite prior steroids; and the bone-protection dilemma of high-dose steroids after a prior atypical femoral fracture.

**Case description:**

An 86-year-old woman described three weeks of bilateral temporal headache and jaw claudication, followed two weeks later by sudden painless left visual loss (a curtain), with acuity reduced to hand movements; she then noticed right-eye scotomata. Her background included osteoporosis and a prior left femoral fracture regarded as a possible atypical femoral fracture from long-term bisphosphonate therapy, with hypertension, hypercholesterolaemia and asthma.

Before the diagnosis was reached she had a two-week wait for a primary-care appointment, an emergency attendance where CT head excluded stroke, and an intervening bank-holiday period, after which ophthalmology suspected GCA and referred her acutely. The left temporal artery was tender, with both temporal arteries later palpable.

CRP was 123 mg/L and ESR 62 mm/h. Colour Doppler ultrasound of the temporal and axillary arteries, performed after glucocorticoids had begun, showed diffuse intima-media thickening of both common superficial temporal arteries and their frontal and parietal branches (left worse than right), with a halo sign and a positive compression sign; the axillary arteries were normal, indicating no large-vessel involvement. Left temporal artery biopsy showed lymphocytic infiltration of the arterial wall with histiocytes forming a probable loose granuloma and fragmentation of the internal elastic lamina, confirming arteritis.

She received intravenous methylprednisolone 1 g daily for three days, then oral prednisolone 60 mg daily, with a proton-pump inhibitor, calcium and vitamin D, and aspirin. At four weeks her headache and jaw claudication had resolved and inflammatory markers had normalised (ESR 5 mm/h, CRP 2.1 mg/L); left vision did not recover, but the right eye was preserved.

**Discussion:**

Three lessons emerge. First, the case shows how readily GCA-related sight loss becomes established once recognition is delayed. Cranial symptoms preceded visual loss by some weeks, and the diagnosis was reached only after several points of contact, by which time ischaemia was irreversible. Established arteritic anterior ischaemic optic neuropathy rarely reverses, so the priority is early recognition to prevent visual loss and, once treatment begins, to protect the fellow eye. Observational fast-track cohorts have reported falls in permanent visual impairment (for example from roughly 37% to around 9%), though these figures are uncontrolled.

Second, bilaterality should be read as severity, not rarity. Bilateral temporal halo occurs in roughly 40 to 50% of GCA, so it is not itself unusual; a greater inflammatory burden instead signals higher ischaemic risk. Notably, the halo remained demonstrable despite steroids having been started. Because the temporal halo can fade within days of glucocorticoids, imaging should be prompt but must never delay treatment, and a negative scan would not have excluded GCA. Biopsy provided confirmatory tissue, although a clearly positive ultrasound is often treatment-decisive on its own.

Third, bone protection posed a genuine dilemma. Prolonged high-dose glucocorticoids conferred very-high fracture risk, yet a prior bisphosphonate-associated atypical femoral fracture argued against re-exposure to antiresorptives. This favoured an anabolic-first strategy (for example teriparatide) with urgent bone-health review, while steroid-sparing therapy such as tocilizumab could reduce cumulative glucocorticoid exposure and thereby skeletal harm. The case illustrates individualised prescribing at the intersection of vasculitis and metabolic-bone guidelines.

**Key learning points:**

1. Treat suspected GCA as an ophthalmic emergency: start high-dose glucocorticoids immediately to protect the fellow eye, because established arteritic anterior ischaemic optic neuropathy almost never recovers and no investigation should delay treatment.

2. Fast-track temporal artery ultrasound is first-line: a bilateral halo with a positive compression sign is highly specific and remains demonstrable in the first days after steroids, so image promptly but never defer treatment for it.

3. Recognise GCA early to protect sight: cranial symptoms often precede visual loss, so prompt recognition, same-day treatment and clear referral pathways are the key modifiable factors in preventing irreversible ischaemia.

4. Individualise bone protection: prolonged high-dose steroids confer very-high fracture risk, but a prior bisphosphonate-associated atypical femoral fracture contraindicates antiresorptives, favouring an anabolic agent such as teriparatide plus steroid-sparing tocilizumab to limit cumulative glucocorticoid exposure.

